# Fast and accurate assessment of depression based on voice acoustic features: a cross-sectional and longitudinal study

**DOI:** 10.3389/fpsyt.2023.1195276

**Published:** 2023-06-21

**Authors:** Yang Wang, Lijuan Liang, Zhongguo Zhang, Xiao Xu, Rongxun Liu, Hanzheng Fang, Ran Zhang, Yange Wei, Zhongchun Liu, Rongxin Zhu, Xizhe Zhang, Fei Wang

**Affiliations:** ^1^Psychology Institute, Inner Mongolia Normal University, Hohhot, Inner Mongolia, China; ^2^Early Intervention Unit, Department of Psychiatry, The Affiliated Brain Hospital of Nanjing Medical University, Nanjing, China; ^3^Functional Brain Imaging Institute, Nanjing Medical University, Nanjing, China; ^4^Laboratory of Psychology, The First Affiliated Hospital of Hainan Medical University, Haikou, Hainan, China; ^5^The Fourth People’s Hospital of Yancheng, Yancheng, Jiangsu, China; ^6^School of Biomedical Engineering and Informatics, Nanjing Medical University, Nanjing, China; ^7^College of Medical Engineering, Xinxiang Medical University, Xinxiang, Henan, China; ^8^School of Computer Science and Engineering, Northeastern University, Shenyang, Liaoning, China; ^9^Department of Psychiatry, Renmin Hospital of Wuhan University, Wuhan, Hubei, China

**Keywords:** depression, voice acoustic features, deep learning, Internet-based cognitive-behavioral therapy, cross-sectional, longitudinal

## Abstract

**Background:**

Depression is a widespread mental disorder that affects a significant portion of the population. However, the assessment of depression is often subjective, relying on standard questions or interviews. Acoustic features have been suggested as a reliable and objective alternative for depression assessment. Therefore, in this study, we aim to identify and explore voice acoustic features that can effectively and rapidly predict the severity of depression, as well as investigate the potential correlation between specific treatment options and voice acoustic features.

**Methods:**

We utilized voice acoustic features correlated with depression scores to train a prediction model based on artificial neural network. Leave-one-out cross-validation was performed to evaluate the performance of the model. We also conducted a longitudinal study to analyze the correlation between the improvement of depression and changes in voice acoustic features after an Internet-based cognitive-behavioral therapy (ICBT) program consisting of 12 sessions.

**Results:**

Our study showed that the neural network model trained based on the 30 voice acoustic features significantly correlated with HAMD scores can accurately predict the severity of depression with an absolute mean error of 3.137 and a correlation coefficient of 0.684. Furthermore, four out of the 30 features significantly decreased after ICBT, indicating their potential correlation with specific treatment options and significant improvement in depression (*p* < 0.05).

**Conclusion:**

Voice acoustic features can effectively and rapidly predict the severity of depression, providing a low-cost and efficient method for screening patients with depression on a large scale. Our study also identified potential acoustic features that may be significantly related to specific treatment options for depression.

## Introduction

Depression is typically diagnosed using self-report scales, which rely on patients’ responses to standardized questions (1). However, the accuracy of this method can be limited by factors such as patients’ self-awareness and truthfulness, as well as social stigmas surrounding mental illness. Lengthy questionnaires may also induce fatigue or impatience. As such, more objective biomarkers of depression are needed to improve diagnosis and assessment (2). Acoustic features have emerged as an important and objective measure of emotion, particularly for depression, as they often exhibit specific acoustic features that provide important cues for clinical identification and diagnosis (3). In the present research on the acoustic features of depression, the majority of studies have focused on the differences in the acoustic features of healthy and depressed people (4, 5), with few studies on the changes in the acoustic features of depressed patients over the course of psychological treatment, which will be the focus of our study.

Emotional states can significantly impact the function and structure of the vocal system, as expressed through rhythm, and prosody of voice (6, 7). According to previous research, positive emotions tend to result in higher pitched and louder voice that is faster, whereas negative emotions are characterized by lower volume, slower voice, and longer pauses (8). In addition to prosodic features, there are also significant differences in frequency spectrum features between positive and negative emotions (9). Specifically, negative emotions tend to exhibit more high frequency sounds, an increase in fundamental frequency (F0) rises, and a decrease in formants compared to positive emotions (10). Furthermore, Mel Frequency Cepstral Coefficients (MFCC) have been identified as potential biomarkers for major depression severity and recovery process (11). Studies have shown that various prosodic features, including pause time and total voice volume, exhibit significant negative correlation with depression severity, and that objective acoustic parameters show a decrease in average weighted variance (AWV) (12). As depression severity increases, the range of voice acoustic variation narrows and the acoustic track becomes smoother. Additionally, other voice acoustic features such as the coefficient of variation (COV) of second formants have been found to be significantly correlated with depression severity (13).

However, previous studies on voice acoustic biomarkers for depression have several limitations, such as use of self-reported symptoms, unstructured voice design, and relatively simple data analysis, which may limit the robustness of the findings (14–16). Furthermore, voice overlap induced by interactive interviews can be an obstacle for standard data processing. To address these issues, the DAIC (Distress Analysis Interview Corpus) data by AVEC (Audio-Visual Emotion Recognition Challenge) performed interactive interviews between virtual agents and patients (17). While this model can exclude emotional interaction between psychotherapy and patients, it may also exhibit other voice issues, such as confusion of voice, changes in the virtual agent’s behavior, and long voice (18, 19). In this study, we have not used interactive interviews for voice capture, instead we have used text reading. A standard text reading ensures that each test participant reads the same content, which reduces interference due to different readers and problems with overlapping voice. Thus, increasing the reproducibility and comparability of the experiment.

Voice acoustic features of depression patients may change with the relief of depression, according to clinical impressions. However, it is unclear whether medication, physical therapy, or psychotherapy can affect voice acoustic biomarkers and their sensitivity to treatment response. For example, men and women with depression may have differential responsivity and tolerability to sertraline and imipramine treatment (20). Recent studies have shown that depressed patients demonstrate less voice pause time and more fluent acoustic expression after treatment with drugs or psychotherapy, indicating potential changes in voice acoustic features associated with improvement of depression (21, 22). Additionally, changes in spectrum characteristics may also be associated with depression improvement (23).

The aim of this study was to investigate the relationship between voice acoustic features and depression using machine learning, and to evaluate the validity of these features in predicting the severity of depression. The study also aimed to explore the correlation between changes in voice acoustic features and improvement of depression before and after Internet-based cognitive-behavioral therapy (ICBT). To achieve these goals, objective voice acoustic features related to depression and those sensitive to psychotherapy were identified. The use of a brief standardized reading instead of a nonstandard long interview facilitated post-data processing, improved standardization of data analysis, and increased the accuracy of the training model. In addition, a predictive model was constructed using machine learning to explore the complex, nonlinear correlation between acoustic features and depression and assess the validity of the model. Finally, the longitudinal design of the study allowed for the investigation of specific and effective voice acoustic features for treatment response to ICBT. The findings of this study have important implications for the early detection of depression based on voice acoustic features.

### Ethics statement

This study was conducted in compliance with ethical standards and was approved by the Ethics Committee of Hainan Medical University (HYLL2020005). Informed consent was obtained from all participants before their participation in the study. Participants had the choice to opt-out of the study if they wished to do so. All participants who completed the assessments were provided with their individual psychometric results. In addition, participants who voluntarily chose to participate in the longitudinal study were offered free ICBT.

## Study one: a cross-sectional study of depression and voice acoustic features

### Methods and material

#### Participants

A total of 47 college students with depression (42 females and 5 males) from Hainan Medical University were recruited for this study through online advertisements. The mean age of the participants was 20.51 ± 1.50 years, and their ages ranged from 18 to 24 years. The inclusion criteria for depression were based on the self-rated Patient Health Questionnaire-9 (PHQ-9), with a total score of 5 or higher for initial screening of depressive symptoms (24). Participants who met the inclusion criteria were then assessed by a standardized HAMD-17 telephone interview conducted by psychiatrists from China Medical University and Hainan Medical College with consistent training. A score of ≥7 on the Hamilton Depression (HAMD) Scale was used to determine depressive symptoms. The measurement of depression in this study was based on recent depressive symptoms rather than individuals who have been clinically diagnosed with severe depression. Participants with a score of ≥3 on item 9 of the PHQ-9 indicating suicidal ideation or behavior, severe or potential mental illness such as schizophrenia or drug abuse, acute respiratory diseases or those receiving antidepressant treatment and psychological therapy were excluded from the study.

### Voice data set

For the voice data set, neutral readings such as “Life like a summer flower” were used for acoustic sampling in accordance with a previous study’s recommendation. The original audio recorder of an Android mobile phone was used to record mp3 and m4a format recording files, which were pretested to ensure uniform format and parameters. The collected audio files were transcoded into wav format using FFmpeg and the sampling frequency was converted to 16KHz. After the data collection, we utilized endpoint detection and normalizing for pretreatment to reduce confounding factors. Endpoint detection was used to identify the beginning and end of each voice sample, and normalization was applied to adjust the volume of each sample to a standardized level.

### Features extraction

Acoustic features were extracted for each voice frame with a duration of 10 ms. A total of 120 features were calculated for each frame, including 74 COVAREP features, 20 MFCC-deltas, 20 MFCC-delta-deltas (25), 5 formants (26), and peak-to-RMS (27). These features were referred to as Low-Level-Descriptors (LLDs). COVAREP features, which include prosodic, voice quality, and spectral features, were calculated by the COVAREP toolbox at a frequency of 100 Hz (28). A detailed list of COVAREP features is provided in Table 1. Peak-to-RMS, a gross indicator of loudness linked to waveform shapes, was calculated on a segmental level and reflected a local loudness metric related to waveform shape across a few pitch periods (with a frame length of 20 ms and a frame shift of 10 ms). The first 5 formants of the frame (with a frame length of 20 ms and a frame shift of 10 ms) were predicted by linear predictive coding. MFCC-deltas and MFCC-delta-deltas of the first 20 Mel cepstral coefficients in each frame (with a frame length of 20 ms and a frame shift of 10 ms) were calculated using the librosa Library (29). These features contain the dynamic information of the spectrum envelope on a frame of voice signal.

**Table 1:** Summaries of features.

Feature Name		Num
F0	Vocal cord vibration cycle	1
VUV	Vector containing the binary voicing decisions	1
NAQ	Normalized amplitude quotient is presented as a method to parametrize the glottal closing phase	1
QOQ	The quasiopen period describes the duration of the glottal flow above 50% of the peak amplitude	1
H1H2	Difference in glottal harmonic amplitude	1
PSP	Parabolic spectral parameter fitting a parabolic function to the low-frequency part of the estimated glottal flow	1
MDQ	The Maxima Dispersion Quotient, is proposed for discriminating breathy to tense voice	1
Peak Slope	Slope coefficient of a regression line fit to local peak by using wavelet analysis.	1
Rd	The Rd. shape parameter of the Liljencrants-Fant (LF) glottal model using the Mean Squared Phase (MSP) method based on MSPD2	1
Rd-conf		1
Creak	Detect creaky voice using acoustic features by an artificial neural network	1
MCEP	Transform the spectrogram into a Mel spectrum through the Mel scale filter bank, and then perform cepstrum analysis	25
HMPDM	Harmonic Model Phase Distortion Mean and Harmonic Model Phase Distortion Deviation are flexible representation of the glottal source based on the short-term statistics of the phase distortion	25
HMPDD		13
Peak-to-RMS	Peak-to-RMS measure reflecting a local loudness metric related to waveform shape across a few pitch periods	1
Formant	Formants refer to areas where energy is relatively concentrated in the sound spectrum	5
MFCC-deltas	Reflecting the dynamic information of the spectrum envelope on a frame of voice signal	20
MFCC-delta-deltas		20

Following the calculation of the 120 LLDs for each frame, we obtained a total of 1,200 HSFs for each recording, by calculating 10 statistics (maximum, minimum, median, mean, variance, kurtosis, skewness, regression slope, regression intercept, regression R2) for each LLD, in order to integrate the multi-frame LLD information and describe the distribution of each LLD in the time dimension (30). The final features were standardized using the Standard Scaler of the scikit-learn library (31).

To prevent overfitting of the neural network model, we aimed to reduce the dimensionality of the features. However, the original feature set contained 1,200 dimensions for each of the 47 samples. To address this issue, we performed Pearson correlation analyses between the 1,200 high-level statistics functions (HSFs) and the HAMD scores while controlling for sex and age as co-variants. We selected only the HSFs that were significantly (*P*<0.01) related to the HAMD score and assigned the remaining features a value of 0. This approach helped to reduce the dimensionality of the feature set while retaining relevant information for predicting depression severity.

### Prediction model based on neural network

In recent years, machine learning techniques such as artificial neural networks (ANNs) have shown great promise in tasks such as prediction and classification using large amounts of data. However, the performance of an ANN heavily depends on its architecture, including the number of neurons, layers, and activation functions, which are usually chosen manually by the user. In this study, we employed a method called Neural Architecture Search (NAS) to automatically discover the optimal ANN architecture for the HAMD prediction task. This approach can effectively reduce the manual effort required to find the best ANN architecture and potentially improve the prediction performance.

In this study, we designed a base neural network using Keras Library (32), which consisted of several fully connected layer networks. To optimize the architecture and parameters of the neural network, we performed a grid search over a set of hyperparameters, including the number of layers L ∈ {1, 2, 3, 4}, number of hidden nodes in each layer N ∈ {16, 32, 64}, activation function F ∈ {‘relu’, ‘softmax’, ‘elu’, ‘selu’, ‘softplus’, ‘tanh’, ‘sigmoid’}, batch size B ∈ {2, 4, 8}, the optimizer O ∈ {‘mse’, ‘sgd’, ‘RMSprop’, ‘Adam’} and the learning rates LR ∈ {0.01, 0.001, 0.0001, 0.00001}. The combination of parameters that resulted in the smallest Mean Squared Error (MSE) on the test set was selected as the optimal configuration for the model. To assess the performance of the model, we used leave-one-out cross-validation.

To identify the most informative features for predicting HAMD scores, we utilized two random forest regression models implemented in Scikit-learn library (33). One model utilized all HSFs as input features, while the other only used significant HSFs identified through the Pearson correlation analysis. The top 10 features were then selected and analyzed to gain insights into their predictive capabilities.

## Study two: a longitudinal study about improvement of depression symptom and voice acoustic features

### Methods and materials

#### Participants

For Study two, participants were required to meet the inclusion and exclusion criteria established for Study One, as well as agree to a four-week treatment schedule and have access to a computer with an internet connection. Of the 47 people with depression who participated in Study One, 18 participants continued the longitudinal study with a mean age of (20.47 ± 1.52) years, including 16 (88.9%) females and 2 (11.1%) males. Seven of the participants declined to participate in the ICBT program, and 22 did not complete the program.

### ICBT programme

The ICBT programme consists of 12 treatment modules, delivered through the ICBT training platform. The self-help ICBT intervention is 20 min for each module, and participants are required to complete 3 modules a week, completing all the treatment content within 4 weeks. All modules were based on the cognitive-behavioral model by Beck et al. (34). Modules 1 and 2 introduced participants to the definition, symptoms, and causes of depression, as well as the basic cognitive model. Modules 3–6 described how to identify cognitive distortions and cope with unhelpful automatic thoughts in daily life. Modules 7–8 mainly focused on behavioral activation and dealing with intermediate beliefs. Modules 9–11 centered on learning about structured problem-solving approaches and core beliefs, while Modules 12 provided a summary of the treatment and relapse prevention. After completing each module, participants were sent homework assignments through the WeChat Subscription platform.

### Data analysis

In this study, a difference analysis was conducted to compare the voice acoustic features before and after treatment. The normality and variance homogeneity of each feature distribution in the dataset were evaluated using the Shapiro–Wilk test (35) and Levene test (36), respectively. The differences between the pre-and post-treatment voice acoustic features were analyzed using the Mann–Whitney U test (37), and the mean and median of the characteristic changes with significant differences were calculated.

## Results

### Acoustic feature analysis

**Figure 1:**
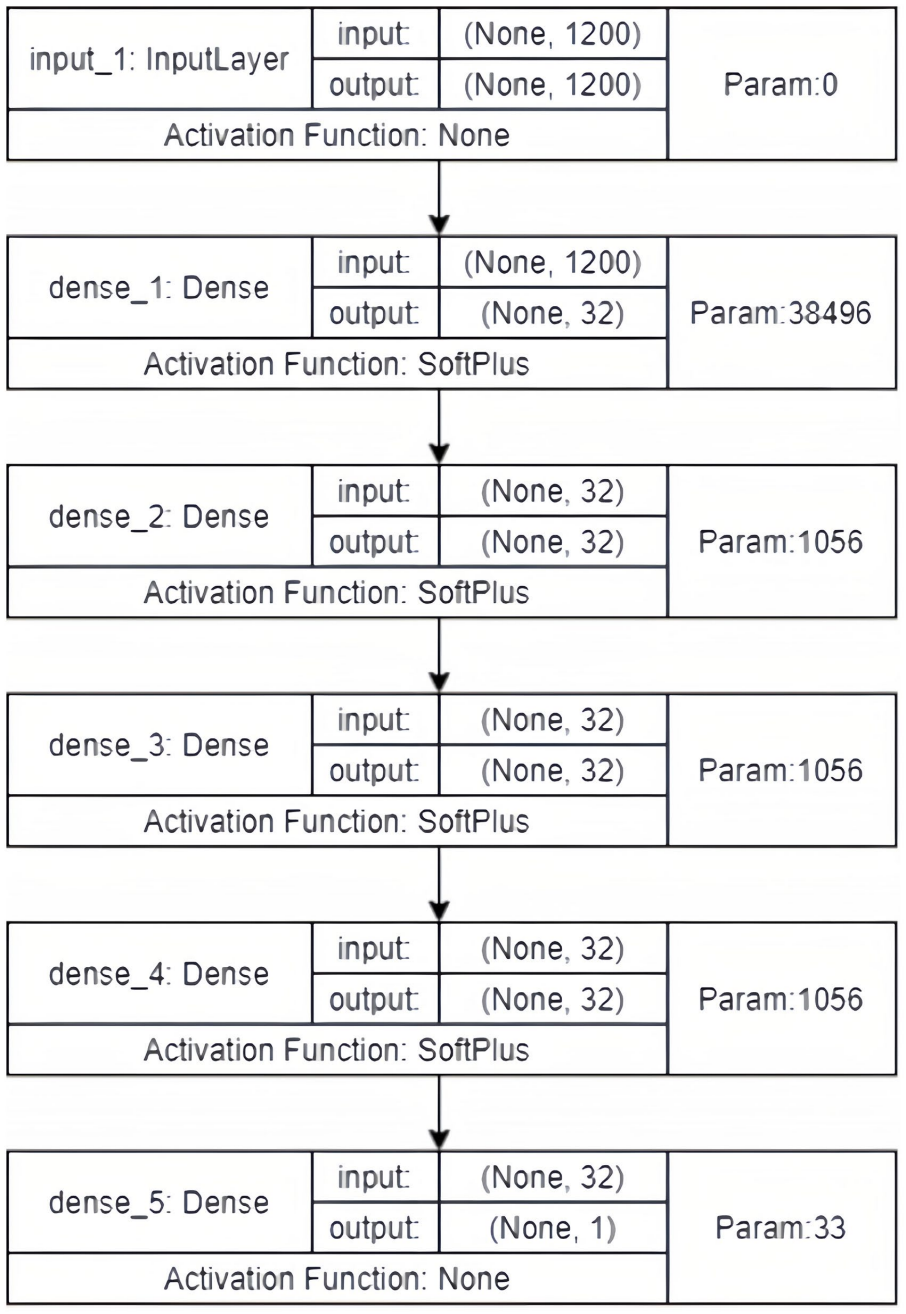
The architecture of the neural network.

We used grid search to obtain the optimal architecture of the neural network, which consisted of 4 hidden layers, each with 32 hidden nodes. The activation function of each layer was softplus. We employed stochastic gradient descent (SGD) as the optimizer and set the learning rate to 0.001 with a batch size of 4 (Figure 1) (38, 39).

**Figure 2:**
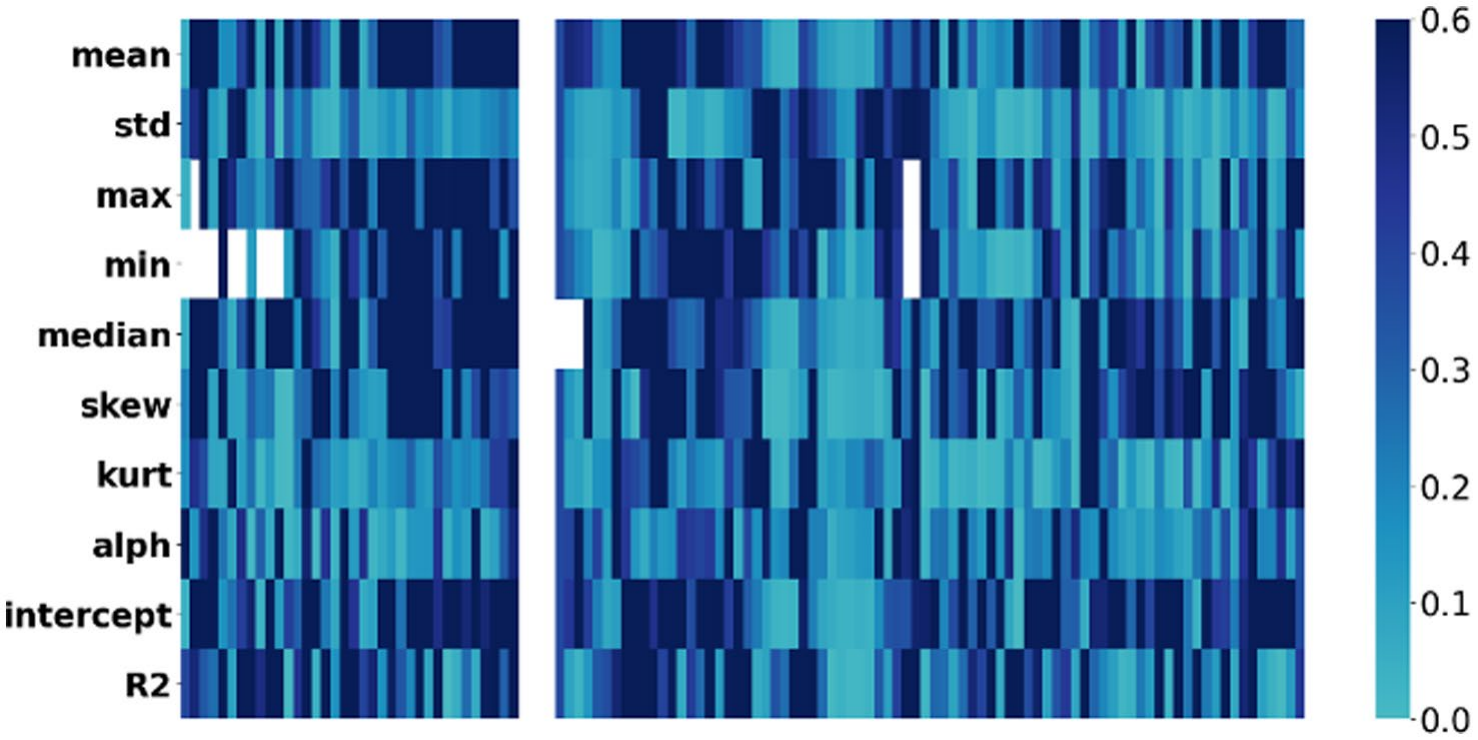
Correlation heatmap: voice acoustic features associated with severity of depressive symptoms. The *x*-axis represents the severity of symptoms, while the *y*-axis represents the voice acoustic features. The colors on the heatmap correspond to the correlation coefficient between symptom and acoustic feature, with blue indicating a lower correlation and black indicating a higher correlation.

**Figure 3:**
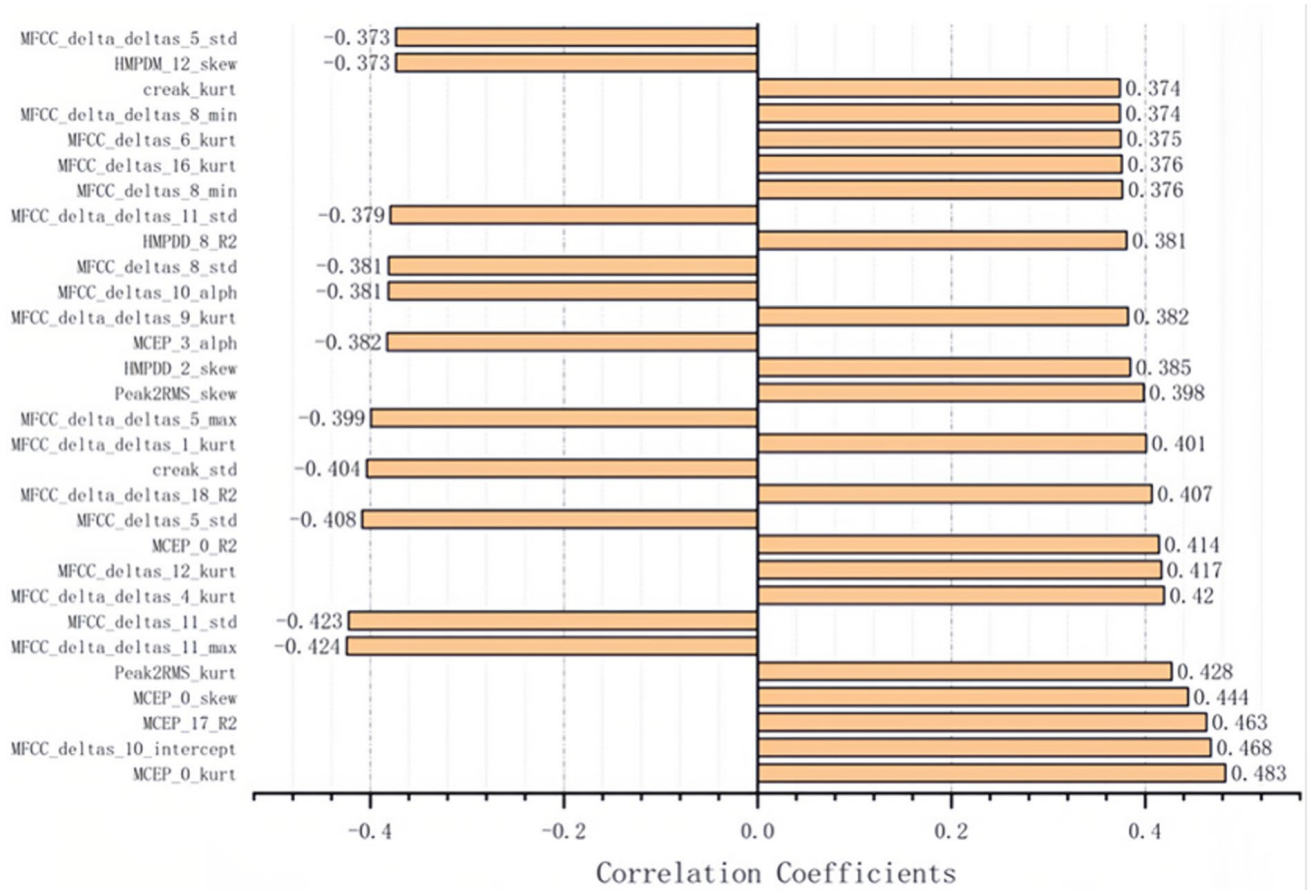
The 30 acoustic features associated with severity of depressive significantly.

We calculated the Pearson correlation coefficient between the 1,200 acoustic features and the HAMD scores, and identified 30 features that were significantly associated with depression severity (*p* < 0.01). These features included Mel-cepstral (MCEP), Mel-scale Frequency Cepstral Coefficients deltas (MFCC-deltas), Mel-scale Frequency Cepstral Coefficients delta-deltas (MFCC-delta-deltas), Harmonic Model Phase Distortion Mean (HMPDM), Harmonic Model Phase Distortion Deviation (HMPDD), creak, and peak to root mean square (Peak to RMS) (Figures 2, 3).

### Prediction model by voice features

**Figure 4:**
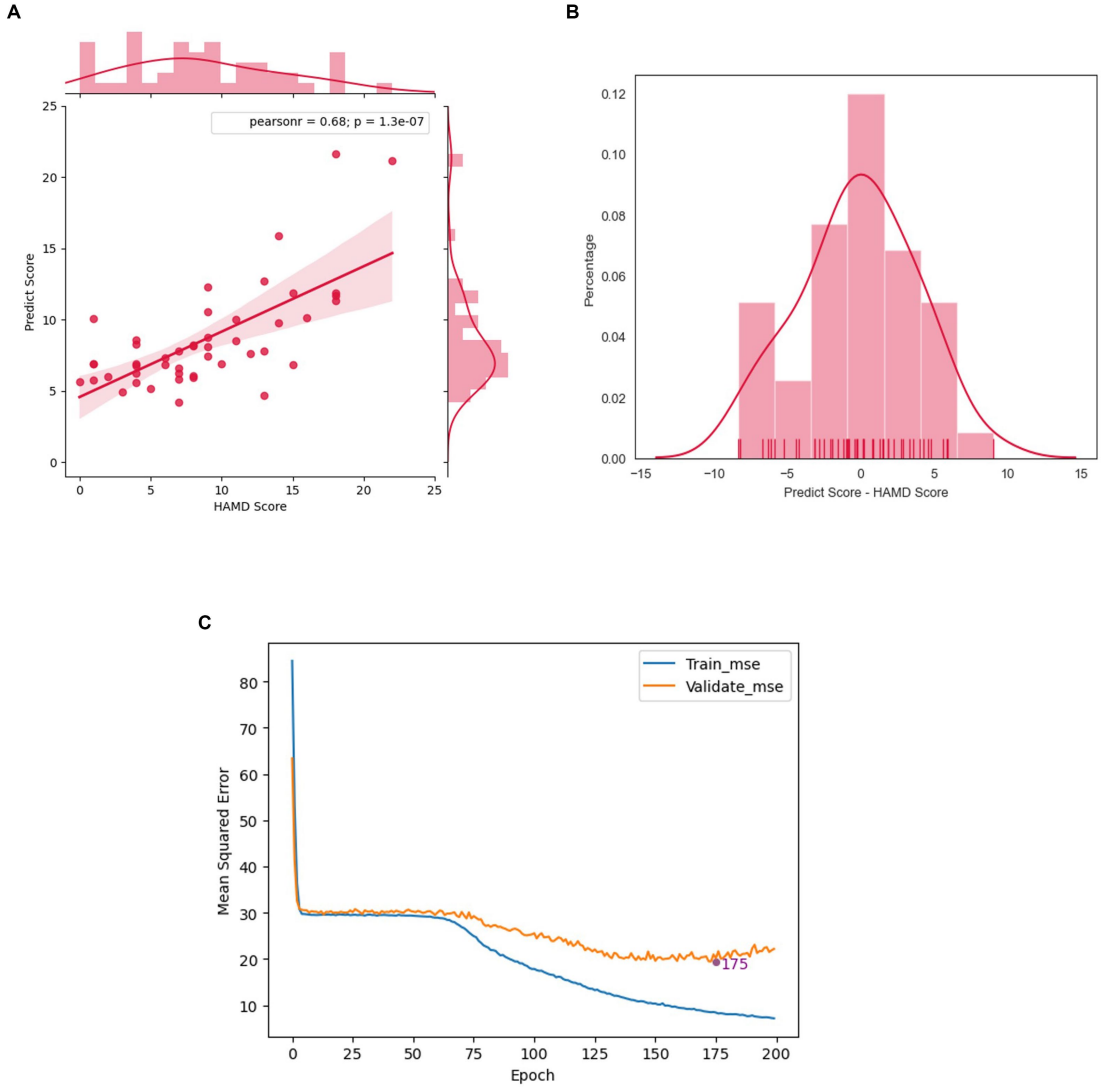
The performance of the prediction model. **(A)** The scatter plot of predicted score and HAMD scores. **(B)** The error distribution of samples. **(C)** Learning curves of our neural network model.

The acoustic features were used to predict the HAMD scores of 47 subjects using the model obtained from the previous analysis with leave-one-out cross-validation. The results showed a strong correlation between the predicted scores and the HAMD scores, with a Pearson correlation coefficient of 0.682 and a *p* value of 1.318 × 10–^7^ (Figure 4A). The error distribution between the predicted and actual HAMD scores is shown in Figure 4B, with a mean absolute error (MAE) of 3.137. This indicates that our model can accurately predict HAMD scores. Moreover, the MAE of 63.83% of subjects was less than 4.0 points, suggesting that most of the subjects’ HAMD scores can be precisely predicted using our model.

To determine the optimal training time and prevent overfitting, we generated a training curve that shows the relationship between Mean Squared Error (MSE) and epochs (Figure 4C). The MSE of the training set decreased as the number of epochs increased. However, the MSE of the validation set reached its lowest point at epoch 175 and then started to increase, indicating that the model began to overfit the data. Therefore, we selected the model at epoch 175 as our final model, as it had the best predictive and generalization abilities.

The performance of our model was evaluated on a workstation with Intel Xeon W-2102 CPU, 8GB RAM and Nvidia GeForce RTX 2080 Ti graphics card. As the prediction of ANN can be done very fast, the runtime of our protocal is mainly determined on the feature extraction. The average runtime to extract the features are listed in Table 2. In general, the whole runtime of the whole process could be done in 347.6 s.

**Table 2:** Time consumption of each step of our diagnosis method.

Step Name	Average time consumption (s)	Standard deviation of time consumption (s)
Recording	100.1	19.3
Extract features using the Covarep toolbox	244.8	48.8
Extract MFCC related features	2.5	0.4
Calculate Peak-to-RMS feature	0.2	0.04
Average consumption of each recording	347.6	

Furthermore, we used random regression forest to identify the most important features in predicting HAMD scores. Table 3 lists the top-10 features for predicting HAMD scores. We found that some acoustic features were repeated, suggesting that they are key factors for prediction in both feature sets. Specifically, four features were found to be important: the regression fit of HMPDD 8, the skewness of MCEP 0, the standard deviation of creak, and the regression intercept of MFCC deltas 10 (Table 3).

**Table 3:** The top 10 feature weights of two random forest regression models separately trained by all features and significant features.

Significant features		All features	
Name	Importance	Name	Importance
**HMPDD_8_R2**	0.107	**creak_std**	0.023
**MCEP_0_skew**	0.096	MFCC_delta_deltas_20_std	0.022
MCEP_17_R2	0.068	Peak-to-RMS_median	0.021
MFCC_deltas_10_alph	0.064	MCEP_9_alph	0.021
MCEP_0_kurt	0.062	**MCEP_0_skew**	0.019
**creak_std**	0.061	**MFCC_deltas_10_intercept**	0.016
**MFCC_deltas_10_intercept**	0.05	HMPDM_8_min	0.015
MFCC_deltas_16_kurt	0.045	MFCC_delta_deltas_4_R2	0.014
MCEP_3_alph	0.036	MFCC_deltas_7_alph	0.014
Age	0.033	**HMPDD_8_R2**	0.014

### Result of longitudinal study

**Figure 5:**
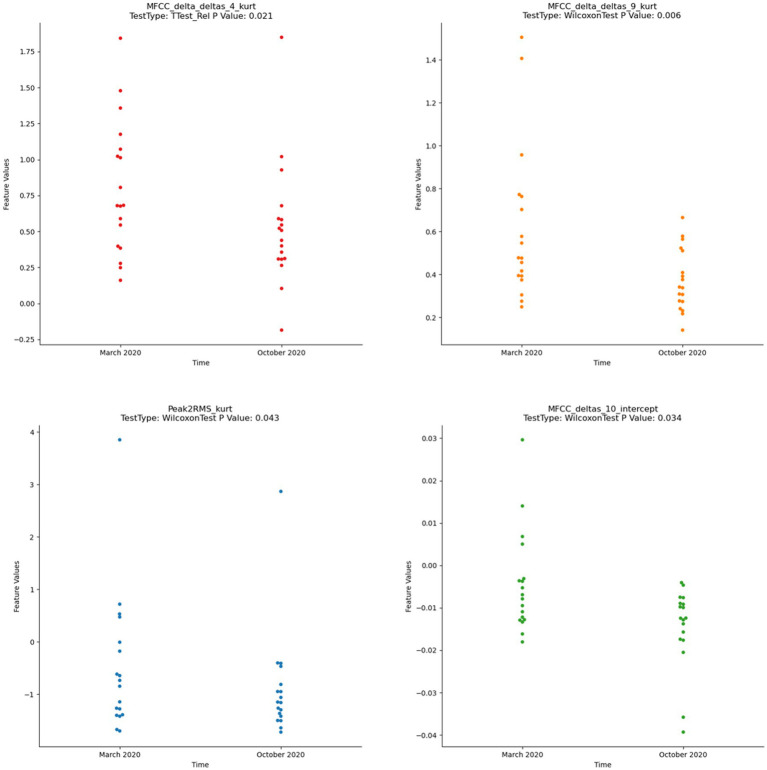
Wilcoxon test of significant difference by voice acoustic features (*p* < 0.05).

All participants who underwent the ICBT program returned to normal scores below the HAMD cut-off, with a mean and SD of 8.79 ± 5.43 and 0.52 ± 0.86 for the pre-and post-ICBT scores, respectively. Among the 30 voice acoustic features analyzed, only nine satisfied both normality and variance homogeneity criteria. Our difference analysis revealed that four voice acoustic features significantly changed in depression participants after ICBT. These four features included Peak2RMS_kurt, MFCC_deltas_10_intercept, MFCC_delta_deltas_4_kurt, and MFCC_delta_deltas_9_kurt (Figure 5). The mean and median of these four features were significantly lower after ICBT, compared to before.

## Discussion

The present study identified 30 voice acoustic features significantly associated with depression, and developed a deep learning model that accurately predicted depression severity. The model also demonstrated good generalization ability and avoided overfitting. These findings suggest that voice acoustic features could serve as objective and effective biomarkers for depression, and be used to monitor treatment response. The longitudinal results showed that four of the voice acoustic features were sensitive to ICBT psychotherapy, indicating that voice acoustic features could potentially be used to monitor treatment progress and adjust treatment plans. Our results are consistent with previous studies that have identified voice acoustic features as reliable biomarkers for depression (2). Overall, our study provides important insights into the potential clinical application of voice acoustic features for depression diagnosis, monitoring, and treatment (12).

Previous studies have mainly utilized difference tests, correlation analysis, and regression analysis to explore depression-related voice acoustic features. However, these methods have limitations in the extraction of relevant quantitative indicators and the accuracy of prediction. In this study, we employed an algorithm to extract more voice acoustic features and related parameters, which enabled us to fully explore depression-related voice acoustic features. By performing dimensionality reduction with a significance level of *P*<0.01, we identified 30 voice acoustic features significantly associated with depression, including loudness, MFCCs, harmonic wave, and creak. These findings were consistent with previous studies that suggested depression can lead to changes in the motor control of the vocal tract, resulting in changes in voice acoustic features such as delayed articulation, dyskinesia, and poor coordination (34). Our study found that Peak to RMS, related to loudness, was significantly associated with severity of depression, which is consistent with previous studies (40, 41). Moreover, MFCCs were also found to be associated with depression severity. Previous studies suggested that MFCCs were associated with less vocal tract changes in depression patients due to the tighter vocal tract caused by psychomotor retardation (42). Creak, which is caused by microtremors of the vocal cords, was identified as a latent biomarker for depression. It was found to increase with the severity of depression and was associated with a higher risk of suicide in depressed patients (43, 44).

In addition, our study explored phase parameters that reflect depression symptoms, which were rarely investigated in previous studies due to the difficulty of extraction (45). We found that fundamental frequency, which responds to the thickness and tightness of the vocal cords, was relatively stable for a few weeks but can manifest lower voice and decreased fundamental frequency in depression (42). Previous studies also demonstrated that the amplitude of the harmonic wave was smaller in depression patients (46). Overall, our study provides valuable insights into the use of voice acoustic features as objective and effective biomarkers for depression diagnosis and treatment response. The comprehensive extraction and analysis of voice acoustic features provide a more accurate prediction model for depression severity, which can improve the accuracy of diagnosis and help develop targeted treatment plans.

To assess the effectiveness of our model, we performed leave-one-out cross-validation on our dataset, which demonstrated that our model was able to accurately predict HAMD scores for most subjects. Compared with previous studies that utilized voice acoustic features to predict PHQ-9 and PHQ-8 scores, our model achieved a smaller mean absolute error and root mean square error, respectively (47). The results suggest that acoustic features may serve as effective external indicators of depression, as they are related to changes in vocal tract status and features. Our study, which utilized a more comprehensive set of voice acoustic features and a deep learning model that accounts for the nonlinear relationship between depression and these features, demonstrated relatively stronger predictive power. Furthermore, based on the top 10 feature weights of random forest regression, creak, MCEP, MFCC, and HMPDD were identified as the most important acoustic features, which are prosodic and spectral features of voice that could serve as decisive biomarkers for depression (48).

Longitudinal follow-up studies have shown that some voice acoustic features not only have predictive power for the severity of depression but also can be associated with the treatment response of ICBT. In this study, the kurtosis of two 20 MFCC-delta-deltas was found to significantly reduce with the improvement of depression in participants who underwent ICBT, and all participants returned to a normal status (49). Previous studies on drug treatment in major depression patients have also reported the normalization of some voice acoustic features with remission of symptoms (41). Therefore, MFCCs could serve not only as a predictor of depression severity but also as potential biomarkers for treatment response. Additionally, the result of this study showed that Peak to RMS, which measures loudness, increased after ICBT, consistent with previous studies. Loudness has been identified as an important biomarker for identifying depression and a sensitive biomarker for the treatment response of psychotherapy in depression, as confirmed by the results of this longitudinal study (12). The sensitivity of vocal acoustic parameters to ICBT may provide a new perspective for optimal treatment options and further confirm the role of direct and indirect acoustic features in identifying depression.

In future studies, we plan to validate our findings and assess potential gender differences in the effectiveness of our intervention by recruiting a more balanced sample of male and female patients. Additionally, we will incorporate other voice tasks and implement a longitudinal follow-up to explore factors that may impact the relationship between vocal features and depression severity. Furthermore, it is worth noting that our study was limited by the absence of a healthy control group. To address this limitation, we will consider including a healthy control group in future studies to better understand the unique acoustic characteristics associated with depression, thus shedding light on the potential diagnostic value of these features.

In this study, we used a machine learning algorithm to accurately extract acoustic feature parameters, allowing for a more comprehensive exploration of the relationship between acoustic features and depression. Furthermore, our use of the random forest regression method to calculate feature weights was a more effective approach than traditional correlation analysis and principal component analysis. We also identified key acoustic features, such as creak, MCEP, MFCC, and HMPDD, as potential biomarkers for depression. Our longitudinal study examining the relationship between acoustic features and treatment response of ICBT provides new evidence for objective identification of depression and assessment of treatment effectiveness. Overall, our findings have significant implications for the use of acoustic features in depression assessment and treatment.

## Data availability statement

The original contributions presented in the study are included in the article/supplementary materials, further inquiries can be directed to the corresponding authors.

## Ethics statement

The studies involving human participants were reviewed and approved by the Ethics Committee of Hainan Medical University (HYLL2020005). The patients/participants provided their written informed consent to participate in this study. Written informed consent was obtained from the individual(s) for the publication of any potentially identifiable images or data included in this article.

## Author contributions

YWa and LL managed the literature searches, participated in the collection and analysis of data, and wrote the manuscript. ZZ, XX, and HF participated in the collection and analysis of data. RaZ, RL, ZL, and YWe gave suggestion for ICBT and research process. FW, XZ, and RoZ designed the study, supervised the sample recruitment, and provided suggestions. All authors contributed to the article and approved the submitted version.

## Funding

This study was funded by Jiangsu Provincial Key Research and Development Program (BE2021617 to FW and XZ), National Science Fund for Distinguished Young Scholars (81725005 to FW), NSFC-Guangdong Joint Fund (U20A6005 to FW), National Natural Science Foundation of China (62176129 to XZ), National Key Research and Development Program (2022YFC2405603 to XZ), Key Project supported by Medical Science and Technology Development Foundation, Jiangsu Commission of Health (ZD2021026 to RoZ), National Natural Science Foundation of China (82151315 to RoZ), Jiangsu Provincial Key Research and Development Program (BE2022160 to RoZ), Inner Mongolia Autonomous Region Postgraduate Education Innovation Program Funding Project (B202101194Z to YW), Hainan Provincial Natural Science Foundation of China (821RC700 to LL), National Key R&D Program of China (2018YFC1314600 to ZL), Henan Province Higher Education Teaching Reform Research and Practice Project (2021SJGLX189 to RL).

## Conflict of interest

The authors declare that the research was conducted in the absence of any commercial or financial relationships that could be construed as a potential conflict of interest.

## Publisher’s note

All claims expressed in this article are solely those of the authors and do not necessarily represent those of their affiliated organizations, or those of the publisher, the editors and the reviewers. Any product that may be evaluated in this article, or claim that may be made by its manufacturer, is not guaranteed or endorsed by the publisher.
